# Women’s Pornography Use Patterns and Sexuality Education in U.S. Public Schools

**DOI:** 10.1007/s10508-024-02905-6

**Published:** 2024-07-12

**Authors:** Julie Fraumeni-McBride, Brian J. Willoughby

**Affiliations:** 1 Attalah College of Educational Studies, Chapman University, Orange, CA 92862 USA; 2 School of Family Life, Brigham Young University, Provo, UT USA

**Keywords:** Sexuality education, Women’s pornography, Women’s sexuality, Women’s sexual health, Abstinence only sexuality education

## Abstract

This study investigated the relationship between sexuality education in U.S. public schools and women's pornography use. Utilizing quantitative methods, we examined a sample of women attending U.S. public schools who reported regular pornography use. Results revealed that, regardless of the type of sexuality education received, women exhibited similar rates of pornography use, with 60% reporting its use. A substantial portion (69%) of the women began using pornography during childhood or adolescence. Women who received abstinence only sexuality education reported higher frequencies of pornography use compared to their comprehensive sexuality education counterparts. About 79% of women using pornography perceived it as a source of sexuality learning, especially regarding sexual pleasure. However, they expressed reluctance in using pornography for sexual education and did not consider it a preferred method for learning about sexuality. The findings suggest the need for comprehensive sexuality education that addresses essential topics, such as sexual pleasure and sexual script development, to cater to women’s diverse learning needs, ideally taught by parents or primary caregivers, but may be necessary for public education in the absence of parental instruction. Policymakers and educators should bridge these gaps to develop more effective sexuality education curricula. This study contributes valuable insights, highlighting the importance of an inclusive approach to sexuality education in U.S. public schools. Future research should explore the implications of different sexuality education approaches on women's sexual development and well-being, emphasizing comprehensive education to foster healthy sexual behaviors among women.

## Introduction

This quantitative study embarks on a distinctive exploration, delving into the intricate relationship between sexuality education and self-reported pornography use. Specifically, the inquiry delves into how women's pornography habits, encompassing both content and frequency, correlate with the sexuality education they have received. The aim is to shed light on ways to enhance recommendations for decision and policymakers, with the ultimate goal of enriching the development of sexual scripts.

The study endeavors to elucidate the potential association between women's engagement with pornography and the nature of sexuality education received during their formative years. By scrutinizing both mandated sexuality education and informal avenues of sexual knowledge acquisition, such as pornography exposure, the investigation seeks to identify gaps within the existing sexuality education curriculum. This understanding is crucial for pinpointing areas where improvements are needed and for addressing potential connections between the quality of education and patterns of pornography use.

### Definition of Pornography

In the context of this study, pornography is defined as sexually explicit materials deliberately crafted to elicit arousal, as outlined by McKee et al. (2019). It encompasses visually stimulating content, such as videos, photogaphs, anime, drawings, short literature, audio recordings, or digital graphics, all depicting explicit nudity and sexual acts, with the intent of provoking arousal, including depicting the process of becoming aroused, arousal itself, and orgasm (West, 2022).

Although the concept of pornography might seem straightforward, its definition is often intricate and subjective. Thus, any study addressing this topic must establish clear and precise constructs. In this study, references to pornography pertain to content of a highly erotic nature, primarily intended for masturbation or to stimulate sexual activity. Such content is commonly found on various platforms, including websites, designed to captivate and engage viewers through their allure.

It is important to note that this sexually explicit content can encompass amateur productions, which might not necessarily be created for financial gain. However, it excludes depictions of nudity found in classical artworks or Renaissance pieces, as well as any content that has traditionally been accessible to individuals of all age groups in public libraries or similar public spaces within the USA. While such content might evoke arousal in some instances, it does not fall under the purview of pornography as defined for the purpose of this literature review (Grubbs et al., 2019).

#### Types of Pornography

Within the realm of explicit content, the world of pornography exhibits a diverse range of genres and modes, each holding distinct characteristics and effects on consumers (Kowalewska et al., 2020). However, past research has often neglected to clearly delineate these genres and modes, leaving the term "pornography" as an overarching concept, potentially leading to generalized assumptions about its impact (McKee, 2007; Roller, 2004). The absence of a clear differentiation between modalities and subcategories has hindered the isolation of specific types of pornography, thereby impeding a more accurate measurement of their unique effects (Leonhardt & Willoughby, 2019).

While the assessment of pornography use typically revolves around the frequency and potential compulsivity, aiming to discern problematic usage (largely through self-identification), it is evident that not all forms of explicit content hold the same implications. For instance, the act of viewing child pornography or bestiality vastly differs from engaging with softcore pornography depicting consenting adults (Walton, 2019). This distinction underscores the importance of recognizing the spectrum of modalities and genres/subcategories within the domain of explicit content.

The survey utilized in this study specifically addresses the various modalities and genres/subcategories, allowing for a more nuanced understanding of participants' experiences and preferences (Leonhardt & Willoughby, 2019). By elucidating the complexities inherent in different types of pornography, this research seeks to shed light on the multifaceted landscape of explicit content consumption and its potential impacts.

#### General Definition of Formal Sexuality Education (Public vs. Private)

Formal sexuality education refers to teaching instruction received in an organized and intentional institutional setting with designated time and planning of elements of the content being taught, typically under the direction of state legislation related to such instruction (Ahluwalia, 2018). This instruction includes a designated curriculum covering topics related to sexuality, including, but not limited to, sexual intercourse, masturbation, HIV, sexually transmitted infections (STIs), contraception, pregnancy, and abortion (SIECUS, 2019).

#### General Definition of Informal Sexuality Education

Informal sexuality education is learning about sexuality that can include knowledge obtained about sexuality from informal sources such as friends, family/parents, teachers, and others (Warren & Neer, 1986) in a manner that is not organized or planned, often lacking in scientific support, or not designated by an evidence-based curriculum. This knowledge can be gained by asking questions and may often result in viewing pornography to learn about sexuality (Litsou et al., 2020).

### Types of Formal Sexuality Education: Abstinence Only and Comprehensive

Different regional territories or states in the USA have differently funded and approved forms of mandated formal sexuality education. The USA has struggled to implement comprehensive sexuality education (CSE) because of the politicization of sexual health conflated with moral incongruence (SIECUS, 2019). For example, some—historically Republican—states require abstinence only sexuality education (AOSE) or heavily incentivize and allocate funding for such education; other—historically Democratic—states mandate HIV/STI prevention sexuality education, with several more progressive states requiring CSE (SIECUS, 2019). The Centers for Disease Control and Prevention’s School Health Profiles data have been consolidated to identify what areas of sexual health are or are not being addressed across the USA (CDC, 2020). Currently, 29 states and the District of Columbia mandate sexuality education, and 16 states require instruction on condoms or contraception when sexuality education or HIV/STI instruction is provided. In addition, 15 states do not require sexuality education or HIV/STI instruction to be age appropriate, medically accurate, culturally responsive, or evidence based/evidence informed. Nine states require sexuality education or HIV instruction to include information on consent, and only eight states require culturally responsive sexuality education and HIV/STI instruction. Seven states have policies including affirming sexual orientation instruction on LGBTQ identities or discussion of sexual health for LGBTQ youth, and nine states require instruction excluding LGBTQ-inclusive language from instruction (SIECUS, 2019).

#### Abstinence Only Sexuality Education

According to the SIECUS (2019), AOSE focuses on waiting to have sex until marriage and usually does not include information on birth control, abortion, STIs, or inclusive sexuality education related to LGBTQ populations. It does not explore sexuality, other than preparing individuals to abstain from sexual intercourse or oral sex of any kind until marriage, as the safest and only effective method to prevent STIs and pregnancy (SIECUS, 2019).

The focus of HIV/STI prevention sexuality education is how to prevent STIs/diseases. This type of education focuses on using protection such as condoms, identifying different types of STIs/diseases, and knowing how and when to seek treatment and screenings. It also explains options for teen pregnancy, such as abortion and other contraceptives (SIECUS, 2019). Abstinence may be covered, but the focus typically is on specific strategies to prevent unwanted outcomes when engaging in sexual activity.

#### Comprehensive Sexuality Education

CSE is inclusive of other methods for teaching sexuality while incorporating current, relevant, and scientifically supported teachings related to sexuality. The most effective form of sexuality education provides medically accurate information about sexuality that is also inclusive of LGBTQ populations (Grossman et al., 2014). This form of sexuality education focuses on understanding abstinence, HIV/STI prevention (e.g., using condoms), forms of STI/diseases, and when to seek medical attention and treatment. It also includes components of how to have sex safely, and many programs include components of consent.

Few other states have such formalized and detailed requirements for CSE, with California being the first and New Jersey and Oregon following in the requirement to mandate that only medically accurate information be provided as well as inclusion of instruction related to healthy relationships and consent. Only seven states have policies including affirming sexual orientation instruction for LGBTQ identities or discussion of sexual health for LGBTQ youth (SIECUS, 2019).

### The Current Study

A deeper comprehension of the interplay between sexuality education and pornography consumption holds importance. An enhanced grasp of this relationship equips policymakers and decisionmakers with insights essential for refining curriculum and implementing preventive measures. Such enhancements have the potential to empower girls and women by fostering a shift away from excessive reliance on pornography as a source of sexual knowledge. Instead, informed by scientifically grounded instructions, individuals can cultivate healthier sexual and relational attitudes, paving the way for more positive outcomes in terms of sexual health and overall well-being.

The complex relationship between sexuality education and self-reported pornography use remains inadequately understood. Given the potential for problematic pornography use or the interference of pornography on a person’s life- either perceived or actual, particularly among adolescents, investigating this connection is crucial (Fraumeni-McBride, 2019). As comprehensive sexuality education (CSE) has shown positive impacts on sexual health outcomes, the study hypothesizes that it might also reduce negative aspects of pornography use (e.g., problematic pornography use). Notably, existing curricula rarely address pornography, indicating a gap that could benefit from attention (Gouvernet et al., 2017).

The study recognizes that existing research on pornography use is often viewed through preconceived lenses, influencing perceptions (Grubbs et al., 2019). Furthermore, limitations in methodologies, whether quantitative or qualitative, pose challenges such as self-reporting biases (Duffy et al., 2016). Gender biases are prevalent, disproportionately focusing on male perspectives, thus overlooking potential gender-specific differences in pornography experiences (Lefkowitz et al., 2014).

This investigation aims to fill gaps by scrutinizing the interplay between formal sexuality education and pornography use, particularly for women. Hypotheses were designed to test this relationship:

#### H1

Comprehensive sexuality education recipients will report lower frequency of hardcore pornography use compared to abstinence-only education recipients.

#### H2

Comprehensive sexuality education recipients will report lower frequency of all pornography types compared to abstinence-only education recipients.

The study's outcomes are expected to contribute to the understanding of female pornography use and guide curriculum development, aiding public health and education policymakers. Understanding hardcore pornography use relationships as opposed to softcore pornography will allow for analyses to see if severity of pornography use is relevant to any particular factors as hardcore pornography use would suggest an intensity in graphics of explicit content being viewed. Although hardcore pornography use does not exclude softcore pornography from having any relationships with patterns of use and sexuality education, distinguishing the more severe classification of pornography delineated a separation between pornography that would be less subjective in being disputable for classifying as pornography, since some people may not consider softcore pornography to be pornographic.

Ultimately, the study seeks to inform future research, curriculum formation, and policy decisions in the field of sexuality education, providing valuable insights for U.S. public school systems.

## Method

We conducted a cross-sectional study to examine the link between self-reported pornography use and sexuality education in women. While previous research has indicated potential connections between the two, prior studies often lacked substantial population samples for comprehensive results (Kowalewska et al., 2020; Litsou et al., 2020). To address this, we utilized a quantitative survey approach to gather extensive data, allowing for the exploration of self-reported pornography use and sexuality education among women. Descriptive statistics were employed to outline sample characteristics, and assumptions were validated before conducting hypothesis testing using Welch’s *t*-test and ANCOVAs to assess possible covariate interactions. It is important to note that the analysis plan focused on association rather than causation.

### Participants and Procedure

Adult participants, aged 18–34 years, were recruited through Qualtrics Online Panels (Chandler et al., 2019) and compensated at standard rates. The survey, titled "Survey of Personal Attitudes and Experiences," aimed for a randomized sample by not explicitly focusing on pornography. Of the 920 women initially recruited, 866 met all criteria. Selection criteria included living in the USA, attending US public schools, and identifying as female. Among them, 515 reported pornography use, forming the primary sample for hypothesis testing. A larger sample was used for descriptive statistics. The decision to not stratify collection allowed for a more comprehensive understanding of women's sexuality education and pornography use in the USA. The final samples, totaling 866 women in the sample and 421 women out of the total sample who report using pornography, provided sufficient statistical power (Altunışık et al., 2004; Andrade, 2020).

The survey was advertised with the title “Survey of Personal Attitudes and Experiences” to ensure that someone would not select the survey because of a heightened interest in pornography, enabling a more randomized sample to be collected. Once a prospective survey taker selected the survey after clicking on the advertisement, participants read a consent form providing more details about the content of the study, including that the survey contained items related to sex, pornography, illegal activity, masturbation, relationship status, and mental health. Participants were then given the option to continue with the survey or exit. Participants were not required to sign an informed consent form; a passive consent form was presented, and participants’ choice to continue with the survey indicated their consent to all terms of the consent form and acknowledgment of risks and benefits. Participants completed the survey after reviewing the passive consent information presented. Survey questions were displayed in categories, starting with demographics and then questions from the four instruments. The sample was restricted to include only participants who completed the full survey and passed all attention and data quality checks. Attention check questions gaging whether participants answered survey questions conscientiously were located randomly throughout the survey.

### Measures

#### Survey Instruments, Demographic Information, and Additional Items Added by the Researchers

Four survey instruments (not including demographics and supplemental questions) were used in this study as the primary measures. Table 2 provides the name of the instruments used, the author(s) and year(s) the instruments were published, and whether the entire instrument was used or if the instrument was modified. Though only three instruments were used in the data analysis of the hypotheses, participants took a larger survey that also included questions related to demographics and covariates, and other areas of peripheral interest as part of the data collection process (Table 1).

**Table 1:** Demographics

Demographic	# of respondents	%
Age in quartiles (in years)		
20–24	234	27.02
25–28	215	24.83
29–31	210	24.25
32–34	206	23.79
Race		
White	656	75.75
Black or African American	122	14.09
Asian	22	2.54
American Indian, Alaska Native, Native	22	2.54
Mixed race	44	5.08
Socioeconomic status		
Less than $15,000–$30,000	298	34.41
$30,001–$75,000	393	45.38
$75,001–More than $135,001	175	20.21
Marital status		
Divorced/widowed	46	5.31
Married	257	29.68
Never married	563	65.01
Education level		
HS/GED or less	264	30.48
Bachelors, associates, or some college	538	62.12
Graduate degree	64	7.39
Sexual orientation		
Bisexual	153	17.67
Completely or mostly homosexual/gay/les	49	5.66
Completely or mostly heterosexual/straight	664	76.67
Relationship orientation		
Monogamous	814	94
Polyamorous	52	6
Age at first exposure to pornography		
4–8	49	5.66
12–15	315	36.38
16–18	218	25.17
19–22	74	8.55
23–30	20	2.32
Do you use pornography?		
No	351	40.53
Yes	515	59.47
Using hardcore pornography	371	72.2
Using illegal forms of pornography	107	20.78
Using bestiality	96	18.64
Using child pornography	84	16.31

The two instruments identified in Table 2 are described briefly in the following sections.

**Table 2:** Instruments used in the study

Instrument title	Author(s) and date of publication	Instrument used in full or modified
Pornography Use Measure (PUM)	Leonhardt and Willoughby (2019)	Modified version
U.S. state in which sexuality education received	Survey item requesting state in which respondent lived during high school and received sexuality education	In full

#### Pornography Use Frequency, Category, and Type of Sexuality Education Received

##### Pornography Use Measure (PUM)

PUM was utilized to assess various types of pornography due to the disparity in their relationships on individuals. Initially devised by Willoughby and Busby (2016), the scale was later refined by Leonhardt and Willoughby (2019) and ultimately validated by Busby et al. (2020). Despite the subsequent validation in 2020, the Leonhardt and Willoughby (2019) version was chosen for the current study, primarily due to its alignment with the study's scope.

The scale developed by Willoughby and Busby (2016) and adapted by Leonhardt and Willoughby (2019) employed confirmatory factor analysis to differentiate between the use of sexually provocative media and pornography. This distinction is crucial given the divergent interpretations of pornography. The scale encompasses 22 statements regarding the types of pornography consumed over the previous year, rated on a 6-point scale (1 = None, 6 = Every day or almost every day), an example item being “A video of two men having sexual intercourse with each other.” Notably, seven items from the scale were employed in my study to assess hardcore pornography use, following established operational definitions.

For the study's purpose, hardcore pornography was measured through seven items, all adhering to the operational definition. These items included explicit descriptions and visuals of sexual acts, engaging with homosexual and heterosexual content. Two additional items, considered illegal acts, were excluded from Hypothesis 1 testing but examined separately for descriptive purposes. Participant responses were averaged to determine composite scores, representing the dependent variable for Hypothesis 1 testing.

##### Type of Mandated Sexuality Education Received

Participants indicated their U.S. state of receiving sexuality education, with each state coded based on its prevailing mandated curriculum during their enrollment in public high schools (SIECUS, 2019), categorized into CSE, AOSE, no required curriculum, and/or HIV/STI prevention sexuality education.

Initially, we aimed to employ the Fresno Student Sex Education Survey, designed by the ACLU of California for the Fresno school district, to assess received sexuality education. However, due to its lack of reliability and validity testing, we opted to use participants' state of residence during high school attendance to ascertain mandated curriculum type. Determinations about participants' mandated sexuality education were based on historical records aligned with their estimated high school years (ages 14–17) (SIECUS, 2019), ensuring consistent categorization.

Out of the data, four categories of sexuality education emerged: HIV only (*n* = 3), no mandated curriculum (*n* = 89), CSE (*n* = 60), and AOSE (*n* = 363). For hypothesis testing, AOSE and CSE were selected as independent variables due to their prevalence in U.S. public schools. The demographic question about high school attendance State determined participants' categorization into specific sexuality education groups.

### Data Analysis Plan and Statistical Model

Descriptive statistics and internal consistency were used for computing with Stata Statistical Software for all analyses. Assumption testing was performed to assess normality, variance, and independence of the sample. For all three hypotheses, Welch’s *t* test was used. Welch’s *t* test was used with a *p* value of 0.05 for each of the three composite variables accompanying each hypothesis to compare outcomes between CSE and AOSE groups.

Considerations for potential confounding variables that could be associated with hypothesis testing were assessed. It was determined that potential confounding variables may exist. Following the Welch’s *t* test, a more comprehensive analysis (i.e., analysis of covariance [ANCOVA]) was conducted to control for covariates of age and religiosity because both factors have been cited as potential confounds in the literature related to pornography and sexuality (Carroll et al., 2017). For religiosity, Item “I would describe myself as a religious person,” on a Likert scale of 1 to 5, was used to determine religiosity from the nonreligious–nonspiritual scale (NRNSS; Cragun et al., 2015). For age, the age range of 18–34 was treated as a continuous variable. Post-hoc testing was performed only for ANCOVAs that yielded significant results.

#### Statistical Model and Qualification for the Statistic Used for All Hypotheses

Welch's *t*-test, chosen due to unequal group variance, unpaired, and non-normal distribution, was ideal (de Winter & Dodou, 2010). As data were ordinal, Stata's continuous coding for ordinal responses was used for hardcore pornography use, general pornography frequency, and pornography for sexuality learning.

ANCOVA was performed after each Welch’s *t*-test, adjusting for age and religiosity. This approach ensured a comprehensive analysis, compensating for potential confounding.

#### Sample and Inclusion Criteria

The analysis included participants who completed the entire survey, incorporating attention markers. Respondents failing attention checks were excluded, ensuring no missing data. Participants with no required curriculum (*n* = 89) or HIV/STI prevention education (*n* = 3) were excluded from each hypothesis, leaving CSE (*n* = 60) and AOSE (*n* = 363) participants. Hypothesis testing involved women who used pornography (*n* = 515), specifically those who completed essential survey items (*n* = 421). Demographics reporting encompassed all women in the study.

#### Hardcore Pornography Use by Women

##### H1

CSE recipients will report lower hardcore pornography use frequency compared to AOSE recipients.

The operationalized measure was the frequency of hardcore pornography use, assessed through items in the PUM. Each item's Likert scale response contributed to a composite sum for each Welch's *t*-test.

The groups drawn for this analysis consisted of female pornography users: CSE group (*n* = 60) and AOSE group (*n* = 363). Categorization was based on the high school state's mandated sexuality education type. Questions related to the frequency of hardcore pornography use were measured for each type, though frequency was not central to the hypothesis. Participants rated frequency on a scale from 1 (None) to 6 (Every day or almost every day), as depicted in Tables 3, 4.

**Table 3:** Summary of hypotheses, measures, and variables

Hypotheses	Measure name and citation	Variables measured	Analysis approach
Hypothesis 1: Women who received comprehensive sexuality education will use hardcore pornography less frequently than women who received abstinence-only sexuality education	U.S. state where respondent’s sexuality education was received; PUM	Type of sexuality education (abstinence only vs. comprehensive) and frequency of hardcore pornography viewing	Welch’s *t* test
Hypothesis 2: Women who received comprehensive sexuality education will use all pornography types less frequently than women who received abstinence-only sexuality education	U.S. state where respondent’s sexuality education was received; PUS	Type of sexuality education (abstinence only vs. comprehensive) and frequency of pornography viewing	Welch’s *t* test

**Table 4:** Questions related to frequency of hardcore pornography used for each type of hardcore pornography

Question	Content
76.3	A video of two men having sexual intercourse with each other
76.2	A detailed description in writing of a couple engaging in foreplay and sex that includes mention of specific sexual body parts that are touched and aroused as sexual acts are performed
78.1	An image of a heterosexual couple having sex which shows the man’s penis penetrating the woman
78.3	A video showing two naked women or men manually stimulating each other
78.4	A video of a woman or man alone masturbating
78.5	A novel that includes one graphic depiction of sexual intercourse
78.7	A video that graphically depicts a three-way sexual encounter

Select hardcore pornography items of the PUM used for the purpose of this hypothesis with permission from the author

#### Pornography Use Frequency by Women

##### H2

Women who received CSE will exhibit lower frequency of all pornography types compared to AOSE recipients.

To test this, we examined whether receiving CSE (based on the state of education) was associated with lower general pornography viewing frequency (as measured by the PUM) compared to AOSE recipients.

The objective was to ascertain if a significant distinction existed between women who received CSE or AOSE in terms of overall pornography viewing frequency. The analysis included questionnaire respondents who were categorized as receiving comprehensive or abstinence-only sexuality education. The measure was derived from participants' responses to the initial portion of the PUM survey, aggregated into a composite sum variable.

The groups for analysis were sourced from the same sample: CSE group (*n* = 60) and AOSE group (*n* = 363). Participant grouping was determined based on the sexuality education mandated during their high school years in their respective states. The variables assessed focused on the general frequency of pornography use, without specifying particular categories or genres. This set of items captures participants' frequency of pornography use across various contexts and situations.

#### Controls

All analyses controlled for age and religiosity. Previous research has emphasized the significance of attitudes toward pornography as a crucial variable in understanding its usage patterns. This is pertinent because certain correlations between pornography consumption and outcomes might stem from inherent disapproval or moral reservations regarding pornography (Grubbs & Perry, 2019; Willoughby et al., 2016). For religiosity, Item “I would describe myself as a religious person,” on a Likert scale of 1 to 5, was used to determine religiosity from the nonreligious–nonspiritual scale (NRNSS; Cragun et al., 2015). For age, the age range of 18–34 was treated as a continuous variable. Post-hoc testing was performed only for ANCOVAs that yielded significant results.

## Results

### Demographics and Descriptive Statistics

We recruited 920 participants for the survey. After attention checks and quality markers were reviewed for data cleaning, 866 participants met inclusion criteria. Of those 866 participants, 515 participants reported using pornography. Therefore, some analyses included all 866 participants and others included 515 participants; 421 pornography users answered all questions necessary for hypothesis analysis—CSE pornography (*n* = 60) and AOSE pornography users (*n* = 363)—as appropriate for each statistical analysis. For some items, 422 or 421 participants were included depending on accuracy in completing survey items for a specific hypothesis. Demographics are described in detail in Table 1.

### Hardcore Pornography Use

The composite sum of all variables in Welch’s *t* test resulted in the hypothesis being rejected. Results were not significant between the means of the two groups (*t* = 1.43, p = 0.16). Both groups of sexuality education respondents reported using hardcore pornography at similar rates. A key for each item used in the sum of all variables is outlined in Table 3.

Following the Welch’s *t* test, an ANCOVA was conducted. Individuals from the two sexuality education groups (i.e., independent variable) were compared by using an ANCOVA on hardcore pornography use (i.e., dependent variable) while controlling for age and religiosity (i.e., covariates). There was no significant effect of sexuality education type received on hardcore pornography used by women after controlling for the effect of age and religiosity, *F*(1, 418) = 1.22, *p* = 0.27 (see Tables 5, 6).

**Table 5:** Hardcore pornography use Welch’s *t* test statistics

Variable	Abstinence only		Comprehensive		Welch’s *t* test (*df*)	*p* value
	*M*	*SD*	*M*	*SD*		
Hardcore pornography use	15.55	6.80	14.33	6.00	1.43 (86.95)	0.16

For the composite sum variables, the standard *p* value of 0.05 was used

**Table 6:** Hardcore pornography use ANCOVA adjusted for covariates of age and religiosity hardcore pornography use

Variable	*df*	Sum sq	Mean sq	*F*	*p*
Sexed type	1	53.59	53.59	1.22	0.27

### Pornography Use Frequency

Although Hypothesis 2 was similar to Hypothesis 1, Hypothesis 2 was unique as it looked at pornography use frequency more generally, whereas the aim of Hypothesis 1 was to look at hardcore pornography rather than pornography use in general. Although the frequency of pornography use was not the direct aim of measurement for Hypothesis 1, it was used to rule out whether hardcore pornography was being used regularly or habitually versus one time or on a rare occurrence and the frequency of such use was not considered, rather categorized into two distinct categories “use” or “don’t use.” 

The initial Welch’s *t* test on frequency of pornography use resulted in the hypothesis being accepted; statistical significance was found between the means of the two groups, thus supporting the hypothesis (*t* = 2.46, p = 0.02). Women who received CSE reported using pornography in general, less frequently than their peers who received AOSE. Women who received AOSE reported using pornography more frequently than women who received CSE.

Following the Welch’s *t* test, an ANCOVA was conducted. Individuals from the two sexuality education groups (i.e., independent variable) were compared by using an ANCOVA on frequency of pornography use (i.e., dependent variable) while controlling for age and religiosity (i.e., covariates; see Tables 7, 8). There was no significant effect of sexuality education type received on hardcore pornography used by women after controlling for the effect of age and religiosity, *F*(1, 418) = 1.22, *p* = 0.27 (see Table 3, 4).

**Table 7:** Pornography use frequency Welch’s *t* test statistic

Variable	Abstinence only		Comprehensive		Welch’s *t* test statistic (*df*)	*p* value
	*M*	*SD*	*M*	*SD*		
Frequency of pornography use	15.66	5.42	13.98	4.83	2.46 (86.38)	0.02

For the composite sum variables, the standard *p* value of 0.05 was used

**Table 8:** Frequency of pornography use ANCOVA results adjusted for age and religiosity and frequency of pornography use

Variable	*df*	Sum sq	Mean sq	*F*	*p*
Sexed type	1	121.43	121.43	4.29	0.04

## Discussion

### Hardcore Pornography Use

The findings of Hypothesis 1 did not yield significance. Initial Welch’s *t* tests revealed no discernible difference in hardcore pornography use between the CSE and AOSE groups. Subsequent ANCOVA analysis, accounting for age and religiosity as covariates, confirmed no significant difference between the groups, suggesting that age and religiosity were unrelated to hardcore pornography use. This implies that regardless of the type of sexuality education received, women use hardcore pornography at similar rates. Notably, research indicates women generally favor less explicit content (Hald, 2006; Ševčíková & Daneback, 2014), and this study's results align with that trend, indicating similarities between the two groups' tendencies toward hardcore versus softcore pornography viewing. This also corresponds with the prevalence of lesbian pornography use among women, a category often associated with hardcore content (Jones, 2014; Porn Hub, 2021).

Though not factored into hypothesis testing, additional examination of the data suggested that age of initial pornography exposure emerged as an important factor, surpassing other variables in predicting hardcore pornography use, though not included in hypothesis testing. This suggests that early exposure may influence subsequent usage patterns more than the variables measured in the hypothesis testing. Addressing this, comprehensive sexuality education might need to encompass sexuality-related pleasure and the risks of pornography at an early age. The absence of early sexuality education could drive youth toward seeking information from pornography, shaping their sexual scripts (Hussen et al., 2012). Notably, this study examined general hardcore pornography use but did not specifically focus on women's use of lesbian pornography, a prominent category among women. It is plausible that women turn to hardcore pornography, including lesbian content, as a means of exploring female sexual pleasure. This may imply a benefit for identity-specific, pleasure-oriented sexuality education to deter future pornography use that was initiated in childhood.

Though not directly related to the hypotheses, an indirect implication of the study's outcomes highlight the potentially helpful concepts that may inform policymakers on implementing preventative measures against childhood pornography exposure such as earlier and more frequent access to information about sexuality (Hare et al., 2015). Stricter industry regulations, software filtration systems, and early technology safety training should be considered. Simultaneously, developing tailored sexuality curricula that address pornography literacy, identity-based sexual pleasure, and processing early exposure can aid in minimizing the impact of pornography on sexual script development. Policymakers should also assess ways to provide counseling for students exposed to explicit content and offer resources for analyzing and managing the consequences of such exposure. These actions are crucial for safeguarding youth from early exposure and preventing persistent pornography consumption.

The hypothesis's findings highlight the complexities surrounding sexuality education's influence on pornography use. The broader context of early exposure and identity-related education appears to shape usage patterns more significantly. Addressing these factors in comprehensive sexuality curricula and implementing protective measures against early exposure are essential steps in combating the challenges posed by pornography consumption among youth.

### Frequency of Pornography Use

Hypothesis 2 yielded significant results for sexuality education type. Initial Welch's *t* tests demonstrated differences in pornography use frequency between CSE and AOSE groups. ANCOVAs, controlling for age and religiosity, confirmed this significance, attributing the variance to sexuality education type. AOSE recipients reported higher pornography use frequencies than CSE counterparts. This finding underscores the influence of sexuality education on women's pornography consumption, while age and religiosity remain constant.

AOSE's limited curriculum may prompt youth to turn to pornography for comprehensive sexual information, particularly due to its focus on abstinence and exclusion of medically accurate content. Furtherfore, AOSE recipients' higher usage of pornography could stem from unaddressed topics, necessitating further investigation. This suggests cultural conversations influenced by mandated curriculum may impact trust and dialog around sexuality, inadvertently driving women toward more frequent pornography use (Grossman et al., 2014). Consequently, cultural norms, families, and policymakers should encourage open, accurate discussions about sexuality to reduce reliance on pornography (Herbenick, 2012).

The finding that AOSE recipients engage in higher pornography use aligns with Dawson et al.'s (2019a) notion that satisfaction with sexuality education does not necessarily correlate with pornography use. Instead, the education type may dictate consumption frequencies, revealing gaps in addressing healthy sexual development at younger ages. Notably, youth receiving CSE acquire a broader range of sexuality-related knowledge, indicating potential areas that could be addressed in other sexuality education curriculums such as AOSE and ages at which instruction begins, which could suggest an important area for further research to ascertain effective topics of instruction (Hare et al., 2015).

While both education groups exhibited similar patterns of using pornography for sexual pleasure information, the study suggests a potential link between AOSE and elevated use frequencies. This prompts exploration of curriculum components, timing, and content that deter excessive pornography consumption. Cultivating early, accurate, and comprehensive sexuality education might diminish the appeal of pornography as an information source.

The study's outcomes may provide information for policymakers to consider a more consistent frequency in providing sexuality instruction given the frequency that youth are seeking out pornography, suggesting a desire to learn and understand more about their own sexual development. These discussions can prevent adolescents from seeking potentially inaccurate sources, thereby minimizing adverse outcomes like teen pregnancy and STIs (Hussen et al., 2012). Although the recommendation might evoke discomfort, the study's findings may underscore the importance of providing meaningful sexuality education to girls, ultimately curbing potentially problematic outcomes associated with pornography use.

### Implications

#### Sexuality Education Curriculum and Policy

The existing literature underscores the protective effects of comprehensive sexuality education (CSE) against negative sexual health outcomes (Chokprajakchad et al., 2018; Grossman et al., 2014; Leung & Lin, 2019; Oman et al., 2015), despite its limited frequency. However, the impact of sexuality education on pornography use among adolescents remained unclear. The hypotheses were based on the premise that if sexuality education influenced STI prevention, it might also relate to pornography use patterns, shaping long-term sexual scripts (Herbenick, 2012; Wiederman, 2015). Recognizing the absence of mandated pornography discussions, this study assessed the potential influence of sexuality education type on related outcomes.

Significant findings were observed for the frequency of pornography use (Hypothesis 2), highlighting a connection between sexuality education type and usage patterns. Yet, such results were less prominent for hardcore pornography use (Hypothesis 1) and sexual pleasure learning (Hypothesis 3). However, the study reveals that women extensively utilize pornography to comprehend sexual pleasure and engagement, leading to informed sexual script development. Surprisingly, women irrespective of their sexuality education type demonstrated similar rates of hardcore pornography use, suggesting that curriculum components addressing pornography may be lacking.

The study reveals a significant portion of women's exposure to and consumption of pornography begins before adulthood, although the access may be accidental, coerced or intentional, all causes of access would be outside of the girls’ legal consent under the age of 18 and considered a violation just as any types of sexual coercion before the age of consent. The prevalence of pornography access, combined with its unregulated nature, underscores the need for robust sexuality education curricula. Just as previous challenges related to HIV, STIs, and teen pregnancy were addressed through public awareness and comprehensive education, a similar approach can address the complexities of pornography consumption (Hussen et al., 2012). Education emerges as a powerful tool to equip youth with accurate information, curbing undesirable outcomes like early pornography exposure (Blum, 2017).

The research emphasizes that women, regardless of their sexuality education type, consistently turn to pornography for sexual education, uncovering its role as a unique resource. The study emphasizes the urgency of incorporating pleasure into sexuality education, as this aspect remains significantly unaddressed. Offering medically accurate information in curricula could reduce reliance on pornography for sexual knowledge, ensuring healthier sexual script development.

The study suggests that AOSE recipients exhibit higher pornography consumption rates. However, it remains unclear whether this results from content gaps or timing discrepancies. The need for comprehensive sexuality education, encompassing multiple aspects of sexuality may be the most relevant implications of this study. The study's findings highlight the importance of addressing discomfort to promote informed discussions and evidence-based curricula.

In light of these findings, policymakers are urged to consider integrating pleasure-focused education into curricula, limiting reliance on pornography. To counteract the negative influences of pornography on sexual script development, it's imperative to introduce relevant, age-appropriate sexuality education early. Policy discussions and research funding are necessary to develop comprehensive curricula aligned with the needs of young individuals. While more specific relationships and recommendations warrant further research, this study serves as a foundation for guiding policy decisions and advancing sexuality education in public schools.

### Limitations and Future Directions

This study's primary limitations included its cross-sectional nature and reliance on self-reporting, measuring association rather than influence, a common constraint in sexuality education and pornography research. The use of paid Qualtrics respondents might have skewed results. Improved sampling methods are crucial for future studies. The study gathered a wide spectrum of pornography use data; however, focusing on representative categories, including illegal forms, could yield more insightful comparisons. State-level measures, due to recall limitations, substituted individual reports of sexuality education, potentially affecting accuracy.

This research targeted a specific age group (18–34) of women independent use of pornography (without inquiry into coupled pornography use or status of relationship dynamic outside of singledom or marriage), limiting its generalizability. It didn't consider women identifying problematic pornography use, a valuable aspect for further exploration nor did the study explore the extent and nature of access to pornography, as well as the reasons change over time; making links with type of sexuality education received as it only explored women’s current pornography use patterns. Aspects like race, disability, socioeconomic status, education level, and mental health conditions were inadequately represented or assessed. LGBTQ participation was insufficient for a comprehensive analysis, despite being a marginalized group at risk. Moreover, the study didn't directly address mental health and suicidality and the relationship that exists with sexuality and identity, nor did it explore other sexuality learning sources that could be linked to pornography use patterns such as family discussions surrounding the topic, voting tendencies or parenting approaches. Existing surveys lacked female-specific items, suggesting the need for tailored instruments. Variation in mandated sexuality education implementation added a layer of complexity.

Future research avenues include investigating age-appropriate pleasure-focused education for developing girls, exploring motivations behind illegal pornography consumption's impact, and delving into LGBTQ women's experiences with sexuality education and pornography. Racial and ethnic disparities, the fetishization of marginalized groups in pornography, neurodiversity implications, and the incorporation of female sexual pleasure into comprehensive sexuality education warrant further examination. Additionally, the relationships between frequency of pornography use and type of received sexuality education remain essential areas for investigation.

### Conclusions

This study underscores the pressing need for improved sexuality education tailored to the needs of female youth (Chokprajakchad et al., 2018; Grossman et al., 2014; Leung & Lin, 2019; Oman et al., 2015). The alarming exposure of girls to pornography prior to adulthood, notably before adolescence, highlights an educational gap (Lewczuk et al., 2022). This reliance on unregulated sources for sexual information should concern both researchers and policymakers. Regardless of the type of sexuality education, women found pornography useful for learning about sexual pleasure, including related aspects like consent (Quinn-Nilas & Kennett, 2018). Women's interest in gaining knowledge about female-specific pleasure seems to outweigh perceived risks, emphasizing the importance of comprehensive curriculum development. Women who received AOSE viewed pornography more frequently than those who received CSE. Policymakers should focus on inclusive and comprehensive sexuality education that addresses youth interests and needs, aiming to prevent reliance on unreliable sources like pornography. Such education should prioritize sexual consent and safety in the digital age. A comprehensive curriculum should include topics like sexual pleasure, consent, and gender identity to equip youth for healthy sexual development (Farré et al., 2020; Hare et al., 2014, 2015; Mattebo et al., 2012; Ramlagun, 2012; Rothman et al., 2015; Wang & Davidson, 2006). Policymakers need to confront discomfort and prioritize the needs of girls in sexuality education. By bridging the gap between policy and research, we can better support the growth and development of youth, promoting their constitutional right to pursue happiness.
